# Efficacy and safety of praziquantel in preschool-aged and school-aged children infected with *Schistosoma mansoni:* a randomised controlled, parallel-group, dose-ranging, phase 2 trial

**DOI:** 10.1016/S2214-109X(17)30187-0

**Published:** 2017-06-12

**Authors:** Jean T Coulibaly, Gordana Panic, Kigbafori D Silué, Jana Kovač, Jan Hattendorf, Jennifer Keiser

**Affiliations:** a Department of Medical Parasitology and Infection Biology, Swiss Tropical and Public Health Institute, University of Basel, Basel, Switzerland; b Unité de Formation et de Recherche Biosciences, Université Félix Houphouët-Boigny, Abidjan, Côte d'Ivoire; c Department of Epidemiology and Public Health, Swiss Tropical and Public Health Institute, Basel, Switzerland

## Abstract

**Background:**

Praziquantel has been the drug of choice for schistosomiasis control for more than 40 years, yet surprisingly, the optimal dose for children younger than 4 years is not known. We aimed to assess the efficacy and safety of escalating praziquantel dosages in preschool-aged children (PSAC).

**Methods:**

We did a randomised controlled, parallel-group, single-blind, dose-ranging, phase 2 trial in PSAC (2–5 years) and school-aged children (SAC; aged 6–15 years) as a comparator group in southern Côte d'Ivoire. Children were randomly assigned (1:1:1:1) to 20 mg/kg, 40 mg/kg, or 60 mg/kg praziquantel or placebo. Participants, investigators, and laboratory technicians were masked to group assignment, while the investigator providing treatment was aware of the treatment group. The primary objective was to estimate the nature of the dose–response relation in terms of cure rate using the Kato Katz technique. Dose–response curves were estimated using E_max_ models. Available case analysis was done including all participants with primary endpoint data. This trial is registered with International Standard Randomised Controlled Trial, number ISRCTN15280205.

**Findings:**

Between Nov 11, 2014, and Feb 18, 2015, 660 PSAC and 225 SAC were assessed for eligibility; of whom 161 (24%) PSAC and 180 (80%) SAC had a detectable *Schistosoma mansoni* infection. 161 PSAC were randomly allocated of whom 154 received treatment: 42 were assigned to 20 mg/kg praziquantel, of whom 40 received treatment; 38 were assigned to 40 mg/kg praziquantel, of whom 38 received treatment; 41 were assigned to 60 mg/kg praziquantel, of whom 39 received treatment; and 40 were assigned to placebo, of whom 37 received placebo. 180 SAC were randomly allocated of whom 177 received treatment: 49 were assigned to 20 mg/kg praziquantel, of whom 47 received treatment; 46 were assigned to 40 mg/kg praziquantel, of whom 46 received treatment; 42 were assigned to 60 mg/kg praziquantel, of whom 42 received treatment; and 43 were assigned to placebo, of whom 43 received treatment. Follow-up (available-case) data were available for 143 PSAC and 174 SAC. In PSAC, the 20 mg/kg dose resulted in cure in 23 children (62%; 95% CI 44·8–77·5), 40 mg/kg in 26 children (72%; 54·8–85·8), 60 mg/kg in 25 children (71%; 53·7–85·4), and placebo in 13 children (37%; 21·5–55·1). In SAC, the 20 mg/kg dose resulted in cure in 14 children (30%; 95% CI 17·7–45·8), 40 mg/kg in 31 children (69%; 53·4–81·8), 60 mg/kg in 34 children (83%; 67·9–92·8), and placebo in five children (12%; 4·0–25·6). For both age groups, the number of adverse events was similar among the three praziquantel treatment groups, with fewer adverse events observed in the placebo groups. The most common adverse events in PSAC were diarrhoea (11 [9%] of 124) and stomach ache (ten [8%]) and in SAC were diarrhoea (50 [28%] of 177), stomach ache (66 [37%]), and vomiting (26 [15%]) 3 h post treatment. No serious adverse events were reported.

**Interpretation:**

Praziquantel shows a flat dose-response and overall lower efficacy in PSAC compared with in SAC. In the absence of treatment alternatives, a single dose of praziquantel of 40 mg/kg, recommended by the WHO for *S mansoni* infections in SAC can be endorsed for PSAC in preventive chemotherapy programmes.

**Funding:**

European Research Council.

## Introduction

Schistosomiasis is a major public health problem in many parts of the developing world, especially in sub-Saharan Africa. The disease is caused by blood flukes (trematode worms) of the genus *Schistosoma*, with *Schistosoma haematobium, Schistosoma japonicum*, and *Schistosoma mansoni* triggering most infections.^1^, ^2^, ^3^, ^4^, ^5^ Indeed, more than 200 million people are infected globally, with about half of them suffering from morbid sequelae, including haematuria, dysuria, nutritional deficiencies, anaemia, hepatic granulomas leading to (severe) peri-portal fibrosis and consequent portal hypertension, and delayed physical and cognitive development.^6^, ^7^ Praziquantel is the drug of choice for treatment of infections with all *Schistosoma* species in the framework of preventive chemotherapy programmes.^8^ While school-aged children (SAC) are the main target population for treatment, it is becoming increasingly clear that younger children (<6 years) are also affected by schistosomiasis and suffer from morbidity.^9^, ^10^, ^11^, ^12^ Hence, in 2010, WHO recommended inclusion of preschool-aged children (PSAC) in large scale treatment programmes. In the absence of an appropriate paediatric formulation, which is currently under development, broken or crushed praziquantel tablets have been recommended.^13^ Indeed, the efficacy and safety of crushed praziquantel tablets in infants have been assessed in several endemic settings mainly throughout Africa.^11^, ^14^, ^15^, ^16^, ^17^

**Research in context**

**Evidence before this study**

We searched PubMed for studies published before Dec 1, 2016, using the search terms “praziquantel”, “schistosomiasis”, “dose finding”, “school-aged children”, and “preschool-aged children”. Our search identified numerous articles on the use of 40 mg/kg praziquantel in school-aged children, which concluded that this regimen is efficacious and safe. We noted from the year 2000 onwards, there was an increase of studies elucidating the efficacy and safety of crushed praziquantel (40 mg/kg dosage) in preschool-aged children (PSAC). Although this treatment regimen was deemed efficacious and safe, the nature of the dose-related effect has not been studied in both age groups.

**Added value of this study**

The results of our randomised controlled, parallel-group, single-blind, dose-ranging, phase 2 trial study show increasing cure rates and egg reduction rates (ERRs) for school-aged children (SAC) with escalating dosages of praziquantel, while a dose–response relation could not be observed in PSAC using the diagnostic of choice, the Kato-Katz method. The E_max_ model predicted an ERR of 99% at 65 mg/kg in SAC and an ERR of 95% at 50 mg/kg in PSAC, while the ERR of 99% was out of the observed range. Adverse events were mild and transient and included stomach ache, cough, diarrhoea, and vomiting.

**Implications of all the available evidence**

Our dose finding study supports the widely used dose 40 mg/kg of praziquantel for schistosomiasis morbidity control in SAC. Based on our results this dose can also be recommended for PSAC. Drug discovery efforts should be strengthened to have safe and effective alternative treatment options for schistosomiasis available in a timely manner.

Despite the above-mentioned studies, surprisingly, the effective dose for children younger than 4 years is not known. At the moment, praziquantel is widely used off-label at a standard dose of 40 mg/kg to treat PSAC,^18^, ^19^ because this is the recommended dose used for SAC and adults. However, a simple extrapolation of adult praziquantel dosages to children is very uncertain in view of the maturational differences in absorption, metabolism, and elimination.

In more detail, the oral bioavailability of drugs might vary in paediatric and adult populations due to differences in gastric pH and emptying time, intestinal transit time, immaturity of secretion, and the activity of bile and pancreatic fluid. Drug distribution in children and adults differs due to changes in membrane permeability, plasma protein binding, and total body water. Finally, the immaturity of enzyme systems (cytochrome P450), glomerular filtration, renal tubular secretion, and tubular reabsorption in children account for a different excretion of drugs in the paediatric population compared with in adults.^20^, ^21^ Despite this, thorough, quality, dose-finding clinical studies using praziquantel have not been done in PSAC to date.

We aimed to determine the nature of the dose–response of praziquantel in PSAC infected with *S mansoni* to determine the dose of praziquantel that shows an efficacy comparable to the standard dose of 40 mg/kg in SAC in an area where *S mansoni* is endemic.^22^ Our findings, along with concurrent investigations pertaining to the pharmacokinetics of praziquantel in SAC and PSAC (Kovač and colleagues, under preparation) might be pivotal to further optimise the control of schistosomiasis.

## Methods

### Study design and participants

We did a randomised controlled, parallel-group, single-blind, dose-ranging, phase 2 trial was done in five villages located in the health district of Azaguié, southern Côte d'Ivoire. A detailed census was done in November, 2014, which generated lists of PSAC and SAC, including their name, age, and sex. PSAC (aged 2–5 years) and SAC (6-15 years) were enrolled. While the age of SAC was assessed based on their birth certificate at school level, the age of PSAC was confirmed by three potential sources: birth certificate, child's health card—where the date of birth is mentioned, and the verbal statement of the mother in case the two previously mentioned documents were unavailable. The village census identified 141, 167, 139, and 290 PSAC (2–5 years) and 231 SAC (6–15 years). All PSAC (n=737) and SAC registered during the census were invited to participate to the baseline survey.

Ethical approval for the study was obtained by the National Ethics Committee of the Ministry of Health in Côte d'Ivoire (CNER, reference number 037/MSLS/CNER-dkn) and the Ethical Committee of Northwestern and Central Switzerland (EKNZ; reference numner 162/2014).

Community meetings were held to explain the purpose, procedures, potential risks, and benefits of the study. Written informed consent was obtained from parents or legal guardians of participants. SAC were invited to give their assent by writing their name and ticking the following sentence, “I agree to participate in this study” on the assent form. Parents or legal guardians were well informed on the fact that the participation was voluntary hence, they could withdraw their children from the study at any time with no further obligations.

Children were assessed for the presence of an *S mansoni* infection. *S mansoni*-positive children were eligible to participate in this trial. A clinical examination and an oral medical history by active questioning were obtained from all eligible children. Of note, mothers or guardians of the PSAC were asked about the medical history on behalf of their children. Children were excluded and treated with a standard dose of 40 mg/kg praziquantel if they had taken an antimalarial or anthelminthic drug in the past 4 weeks or had any systematic illness—namely, clinical malaria (presence of fever plus positive rapid malaria diagnostic test [ICT Malaria *Plasmodium falciparum* (HRPII), Cape Town, South Africa] according to the national guidelines) or hepatosplenic schistosomiasis. To determine the presence of hepatosplenic schistosomiasis, the extension of the left liver lobe beneath the sternum was measured in centimetres from the mid-sternal line and the extension of the right liver lobe beneath the rib cage was measured in centimetres from the right mid-clavicular line. At the end of the study, 3 months after the inclusion of the first participant, all children enrolled in the study were offered albendazole (400 mg) and praziquantel (40 mg/kg) for the treatment of helminth infections.

### Randomisation and masking

PSAC and SAC with a parasitologically confirmed *S mansoni* infection were stratified by light, moderate, or heavy baseline infection intensities and randomly assigned (1:1:1:1) to placebo or 20 mg/kg, 40 mg/kg, or 60 mg/kg praziquantel using computer-generated stratified block randomisation codes provided by an independent statistician (stratified by two infection intensity strata; block size of eight). Children and laboratory technicians undertaking the diagnostics were masked, while the investigator delivering the treatment was aware of the treatment assignments. Masking was maintained throughout the trial until data cut off. Randomisation codes were released after the database was unlocked.

### Procedures

During the baseline survey, at school level, children were provided with plastic containers labelled with unique identification numbers (IDs) and asked to deliver a fresh stool and urine sample. For the PSAC, mothers or guardians were given the plastic containers labelled with a unique ID and they were asked to obtain a fresh stool and urine sample of their child. From each participating child, two stool samples over two consecutive days and a single urine sample were collected. Stool and urine samples were transferred to a nearby laboratory in Azaguié town and examined on the day of collection. For the diagnosis of *S mansoni*, stool samples were each subjected to duplicate Kato-Katz thick smears (standard template of 41·7 mg).^23^ Eggs of soil-transmitted helminths—ie, *Ascaris lumbricoides*, hookworm, and *Trichuris trichiura*—were also assessed and recorded for each parasite species separately. A subsequent independent quality control of sample results (about 10%) was done. In brief, the result from each slide among the 10% slides is considered correct if the following tolerance margin is not exceeded between the reading of two laboratory technicians: (1) for counts of 100 eggs or less, the difference between technicians' egg counts must not be greater than 10 eggs; and (2) for counts of 100 eggs or more the difference between technicians' egg counts must not be greater than 20 eggs. In case of discrepancy between the results of quality control and the initial reading, all the slides were read once again by the senior technician. Urine samples were subjected to the urine filtration technique for the diagnosis of *S haematobium* using the same quality control process. A commercially available Point-of-Care Circulating Cathodic Antigen (POC-CCA) cassette test (batch number: 34066; Rapid Medical Diagnostics, Pretoria, South Africa) for the diagnosis of *S mansoni* was applied on urine samples from the first day of samples collection. The POC-CCA tests were done and read as described elsewhere.^24^ In addition, a finger prick blood sample was taken, and thick and thin blood smears were prepared for the diagnosis of *Plasmodium* species. Blood smears were stained with Giemsa and examined under a microscope using 100 x oil immersion. The *Plasmodium* density was counted against 200 leucocytes, assuming 8000 leucocytes per μL of blood. If less than 10 *Plasmodium* were found, the reading was continued up to 500 leucocytes. All slides were double-checked by a second laboratory technician and only considered negative if no *Plasmodium* were detected in 100 x oil immersion field by the two independent microscopists. In addition, a rapid malaria diagnostic test was used and the haemoglobin value measured using a calibrated HaemoCue device (HaemoCue 301 system, Ängelholm, Sweden) according to the manufacturer's instructions. Weight was measured in kg using the domestic HAMSON bathroom weighing scale (Graduation increments of 0·1 kg) and height was measured using a common builder's measuring tape.

To assess treatment efficacy, another two stool samples and one urine sample were collected between 21 days and 25 days post-treatment for the follow-up and subjected to the same diagnostic approaches applied at the baseline survey.

### Procedures

After the baseline screening and clinical examination, eligible participants were treated. Before treatment, each participant received breakfast. The breakfast comprised of an equally sized piece of buttered baguette for each child. Praziquantel tablets (600 mg Cesol, kindly provided by Merck (Darmstadt, Germany) or placebo tablets (Fagron, Germany) were given according to the calculated dose per kg of bodyweight in half tablet increments (eg, 1·3 tablets calculated = 1·5 tablets given or 1·2 tablets calculated = 1 tablet given). For PSAC, tablets were crushed using a mortar and pestle and dissolved in a small volume of syrup-flavoured water. SAC and the mothers or guardians of PSAC were interviewed 3 h, 24 h, 48 h, and 72 h after treatment about the occurrence of adverse events and mitigating drugs were provided if necessary. An adverse event was defined as any unfavourable and unintended sign (including an abnormal laboratory finding), symptom, or disease temporally associated with the use of a medicinal or investigational product, whether or not related to the treatment. All adverse event intensities were judged by the study physician, following guidelines by the European Medicine Agency and were graded as mild, moderate, severe, or intolerable.

### Outcomes

Our primary outcome was the cure rate (the percentage of egg-positive children at baseline who became egg-negative after treatment) resulting from different doses of praziquantel based on the Kato-Katz method. The secondary outcomes were egg reduction rate (ERR) and the safety of different doses of praziquantel.

### Statistical analysis

Simulations showed that with 36 children enrolled in each of the four treatment study groups (0 mg/kg, 20 mg/kg, 40 mg/kg, and 60 mg/kg), the dose response prediction model should have a median precision (one half length of the 95% CIs) of 10% points, assuming associated cure rates of 2·8%, 50%, 75%,^25^ and 90%, respectively. The suggested sample size is also in line with the recommendations from Klingenberg in 2009.^26^ To account for losses in the follow-up, the sample size was increased to 40 children in each study group.

Data were double entered into an Excel spreadsheet, transferred into EpiInfo version 3.5.2 (Centers for Disease Control and Prevention; Atlanta, GA, USA) and cross-checked.

Data were analysed with Stata version 13 (Stata Corp; College Station, TX, USA). Participants who had at least one stool sample examined each with duplicate Kato-Katz thick smears at follow-up, were present at treatment day, and not excluded due to a medical condition were included in the final analysis (available case analysis). Imputation of missing data with treatment failure or success was assessed in an intention-to-treat analysis.

Eggs per gram of stool (EPG) were assessed by adding up the egg counts from the duplicate or quadruplicate Kato-Katz thick smears and multiplying this number by a factor of 12 or six, respectively. We classified infection intensity as light (<100 EPG), moderate (100–399 EPG), or heavy (>400 EPG).^27^

Geometric mean egg counts were calculated as:

$${\text{e}}^{1/\text{n}\sum \log(\text{EPG}+1)}-1$$

And the corresponding ERR 100 as:

$$\frac{100-({\text{e}}^{1/\text{n}\sum (\text{EPGfollow-up}+1)}-1)}{({\text{e}}^{1/\text{n}\sum \log(\text{EPGbaseline}+1)}-1)\times 100}$$

A bootstrap resampling method with 5000 replicates was used to calculate 95% CIs for ERRs.

We used an E_max_ model as a primary model to predict the dose–response curves in terms of cure rates and ERRs. The analysis was done with the DoseFinding package (version 0·9–14) of the statistical software environment R (version 3.3.0). E_max_ model was predicted in two stages. For binary data, first cure rates and their covariances were estimated on logit scale via logistic regression. In the second stage, doses and estimated cure rates were fitted to the non-linear E_max_ model to determine the basal effect (treatment effect at dose 0), the asymptotic maximum effect, and ED_50_ (the dose resulting in 0·5 * E_max_). We converted estimates on logit scale to probabilities in the cure rate related dose-response figures.

### Role of funding source

The funder of the study had no role in the study design, data collection, data analysis, data interpretation, or writing of the report. The corresponding author had full access to all the data in the study and had final responsibility for the decision to submit for publication.

## Results

**Figure 1:**
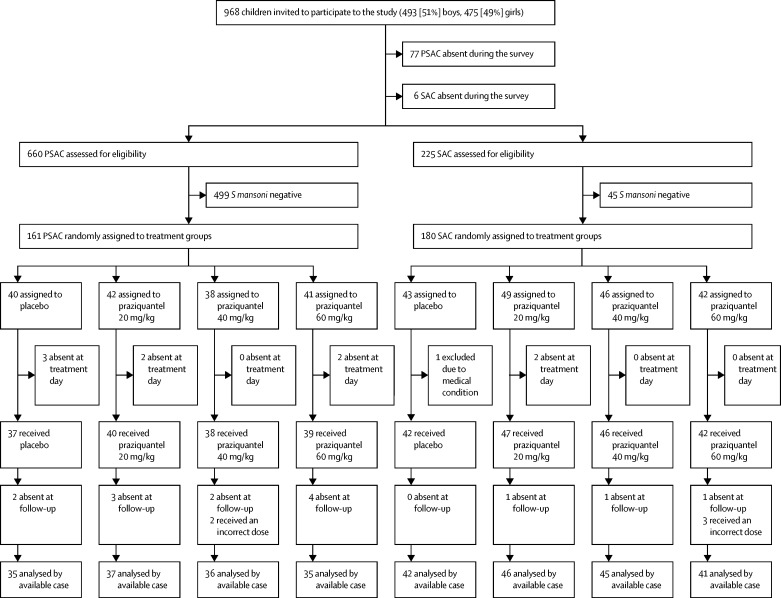
Trial profile PSAC=preschool-aged children. SAC=school-aged children. *S mansoni=Schistosomiasis mansoni*.

Between Nov 11, 2014, and Feb 18, 2015, 968 children were invited to participate in the study. 660 PSAC and 225 SAC participated in the baseline survey (figure 1). Of these, 161 (24%) PSAC and 180 (80%) SAC had a detectable *S mansoni* infection and were randomly assigned to treatment groups. In the PSAC group, 42 were assigned to 20 mg/kg praziquantel, of whom 40 received treatment; 38 were assigned to 40 mg/kg praziquantel, of whom 38 received treatment; 41 were assigned to 60 mg/kg praziquantel, of whom 39 received treatment; and 40 were assigned to placebo, of whom 37 received placebo. In the SAC group, 49 were assigned to 20 mg/kg praziquantel, of whom 47 received treatment; 46 were assigned to 40 mg/kg praziquantel, of whom 46 received treatment; 42 were assigned to 60 mg/kg praziquantel, of whom 42 treatment; and 43 were assigned to placebo, of whom 43 received treatment.

Follow-up (available-case) data were available for 143 PSAC and 174 SAC (figure 1). Of note, 11 PSAC and six SAC provided only a single stool sample at follow-up.

For all treated children, demographic and parasitological baseline data are shown in table 1. The median age, weight, height, and sex of PSAC and SAC were balanced among the treatment groups. At pre-treatment, the geometric mean EPG of faeces ranged between 24·7 and 39·4 in the PSAC treatment groups, and between 71·7 and 84·1 in the SAC treatment groups (table 2). 129 (80%) of PSAC harboured a light *S mansoni* infection versus 93 (52%) of SAC, while about half (87 [49%]) of the SAC had moderate or heavy infection intensities versus 32 (20%) of PSAC. More than half of the SAC were co-infected with *P falciparum* (112 [62%]), while PSAC harboured fewer *Plasmodium* co-infections (53 [33%]). Co-infections with *T trichiura* were common in the study setting, with 56 (35%) PSAC and 97 (54%) SAC co-infected. By contrast, co-infections with *Ascaris,* hookworm, and *S haematobium* were rare, and ranged between 0% and 11% in the different treatment groups. Haemoglobin values were in the normal range with median values between 10·5 and 11·3 in PSAC and between 11·4 and 12·0 in SAC (table 1).

**Table 1:** Baseline characteristics of treatment groups

		**Preschool-aged children**					**School-aged children**				
		Placebo (n=40)	Praziquantel 20 mg/kg (n=42)	Praziquantel 40 mg/kg (n=38)	Praziquantel 60 mg/kg (n=41)	Total (n=161)	Placebo (n=43)	Praziquantel 20 mg/kg (n=49)	Praziquantel 40 mg/kg (n=46)	Praziquantel 60 mg/kg (n=42)	Total (n=180)
Age, years		4 (2–5)	4 (2–5)	4 (2–5)	4 (2–5)	4 (2–5)	8 (6–14)	9 (6–12)	9 (6–13)	9 (6–12)	9 (6–13)
Weight, kg		14 (8–18)	14 (8–18)	14 (11–18)	13 (10–18)	14 (9–18)	23 (15–46)	23 (17–40)	23 (15–41)	24 (16–40)	23 (16–43)
Height, cm		100 (83–105)	95 (77–108)	97 (84–110)	95 (81–105)	96 (80–108)	123 (108–164)	128 (106–152)	126 (106–152)	128 (107–147)	127 (106–156)
Haemoglobin (g/dL)		10·5 (10·3–11·2)*	10·9 (10·3–11·6)†	11·3 (10·2–12·0)‡	10·8 (9·9–11·8)§	10·8 (10·1–11·6)¶	11·6 (10·6–12·6)	11·4 (10·8–12·4)	12·0 (11·3–12·5)	11·6 (10·9–12·9)	11·7 (10·9–12·5)
Sex											
	Girls	17 (43%)	21 (50%)	22 (58%)	20 (49%)	80 (50%)	19 (44%)	26 (53%)	21 (46%)	25 (60%)	91 (51%)
	Boys	23 (58%)	21 (50%)	16 (42%)	21 (51%)	81 (50%)	24 (56%)	23 (47%)	25 (54%)	17 (40%)	89 (49%)
Infection intensity											
	Light	30 (75%)	34 (81%)	32 (84%)	33 (81%)	129 (80%)	23 (54%)	26 (53%)	23 (50%)	21 (50%)	93 (52%)
	Moderate	8 (20%)	6 (14%)	6 (16%)	7 (17%)	27 (17%)	15 (35%)	16 (33%)	16 (35%)	12 (29%)	59 (33%)
	Heavy	2 (5%)	2 (5%)	0	1 (2%)	5 (3%)	5 (12%)	7 (14%)	7 (15%)	9 (21%)	28 (16%)
Co-infections											
	*Schistosoma haematobium*	1 (3%)	0	1 (3%)	3 (7%)	5 (3%)	3 (7%)	3 (6%)	3 (7%)	2 (5%)	11 (6%)
	*Ascaris lumbricoides*	5 (13%)	1 (2%)	0	3 (7%)	9 (6%)	0	3 (6%)	5 (11%)	1 (2%)	9 (5%)
	*Trichuris trichiura*	15 (38%)	15 (36%)	12 (32%)	14 (34%)	56 (35%)	23 (54%)	24 (49%)	27 (59%)	23 (55%)	97 (54%)
	Hookworm	0	1 (2%)	1 (3%)	0	2 (1%)	4 (9%)	3 (6%)	5 (11%)	2 (5%)	14 (8%)
	*Plasmodium falciparum* (based on thin or thick smear)	10 (25%)	17 (41%)	13 (34%)	13 (32%)	53 (33%)	24 (56%)	33 (67%)	28 (61%)	27 (64%)	112 (62%)
	*Plasmodium falciparum*(based on RDT)	10 (25%)	14 (33%)	11 (26%)	13 (32%)	48 (30%)	24 (57%)	32 (67%)	27 (57%)	25 (58%)	108 (61%)

Data are median (IQR) or n (%). RDT=rapid diagnostic test.

* 27 participants.

† 34 participants.

‡ 30 participants.

§ 33 participants.

¶ 124 participants.

**Table 2:** Available case analysis of cure and egg reduction rates of 20 mg/kg, 40 mg/kg, and 60 mg/kg praziquantel versus placebo against intestinal schistosomiasis in PSAC and SAC based on Kato-Katz and POC-CCA

		**Preschool-aged children (PSAC)**				**School-aged children (SAC)**			
		Placebo	Praziquantel 20 mg/kg	Praziquantel 40 mg/kg	Praziquantel 60 mg/kg	Placebo	Praziquantel 20 mg/kg	Praziquantel 40 mg/kg	Praziquantel 60 mg/kg
**Kato-Katz**									
Infected children before treatment		35	37	36	35	42	46	45	41
Cured children after treatment		13 (37·1%; 21·5–55·1)	23 (62·2%;44·8–77·5)	26 (72·2%;54·8–85·8)	25 (71·4%;53·7–85·4)	5 (11·9%;4·0–25·6)	14 (30·4%;17·7–45·8)	31 (68·9%;53·4–81·8)	34 (82·9%;67·9–92·8)
Cured children by infection intensity†									
	Low	11/27	19/27	23/30	21/26	5/24	11/25	16/17	19/20
	Moderate	2/6	4/8	3/6	3/8	0/14	3/16	10/15	9/10
	Heavy	0/2	0/2	0/0	1/1	0/4	0/5	5/13	6/11
Geometric mean EPG									
	Before treatment	40·0	31·7	24·7	39·4	71·7	76·5	84·1	80·2
	After treatment	7·9	2·9	1·3	1·7	31·5	12·1	1·4	0·7
	Egg reduction rate	80·1% (66·3–88·9)	90·7% (82·0–95·7)	94·8% (89·1–98·0)	95·8% (90·2–98·5)	56·0% (36·9–69·7)	84·2% (70·9–91·5)	98·3% (96·7–99·3)	99·1% (97·9–99·8)
Arithmetic mean EPG									
	Before treatment	112·3	140·1	56·5	87·6	179·6	193·7	220·3	292
	After treatment	45·6	26·8	9·0	16·1	132·9	80·2	7·3	29·4
	Egg reduction rate	59·4% (21·6–76·3)	80·9% (56·4–88·6)	84·1% (61·9–94·6)	81·6% (57·1–94·6)	26·0% (0–58·5)	58·6% (24·1–75·9)	96·7% (93·2–98·0)	89·9% (61·9–99·5)
POC-CCA*(with “trace” as positive)									
Infected children before treatment		28	26	25	24	37	42	39	40
Cured children after treatment		6 (21·4%; 8·3–40·9)	4 (15·4%; 4·4–34·9)	8 (32·0%; 14·9–53·5)	8 (33·3%; 15·6–55·3)	4 (10·8%; 3·0–25·4)	8 (19·0%; 8·6–34·1)	15 (38·5%; 23·4–55·4)	22 (55·0%; 38·5–70·7)
**POC-CCA (with “trace'” as negative)**									
Infected children before treatment		24	23	21	23	33	38	35	35
Cured children after treatment		4 (16·7%; 4·7–37·4)	5 (21·7%; 7·5–43·7)	7 (33·3%; 14·6–57·0)	12 (52·2%; 30·6–73·2)	4 (12·1%; 3·4–28·2)	11 (28·9%; 15·4–45·9)	16 (45·7%; 28·9–63·3)	23 (65·7%; 47·8–80·9)

Data are n, n (%), n/N, or n (%; 95% CI). EPG=eggs per gram. POC-CCA=Point-of-Care Circulating Cathodic Antigen cassette test.

* POC-CCA tests were applied on the first urine sample collected from each participant on the first day of collection. Among PSAC and SAC, respectively (seven *vs* five) in placebo, (11 *vs* four) in 20 mg/kg, (11 *vs* six) in 40 mg/kg, and (11 *vs* one) in 60 mg/kg did not provide urine samples on the first day of urine collection

† *Schistosoma mansoni* infection intensity was stratified into low (1–99 EPG), moderate (100–399 EPG), and heavy (≥400 EPG) infection.

**Figure 2:**
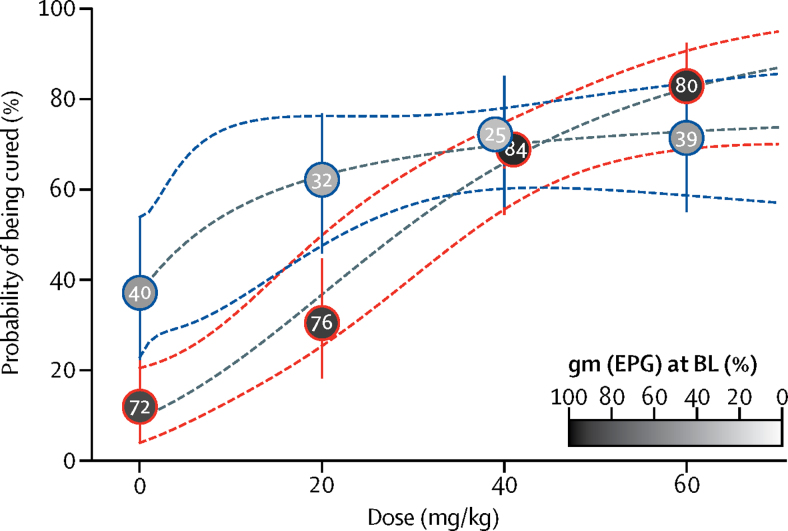
Cure rates in PSAC (blue lines) and SAC (red lines) Circles show observed cure rates with 95% CIs (vertical lines). Numbers and colour code in the circles show geometric mean infection intensities at baseline (BL). Dashed lines represent the estimated dose–response curve and corresponding 95% confidence bands predicted by the E_max_ models. Epg=eggs per gram of stool. PSAC=preschool-aged children. SAC=school-aged children.

**Figure 3:**
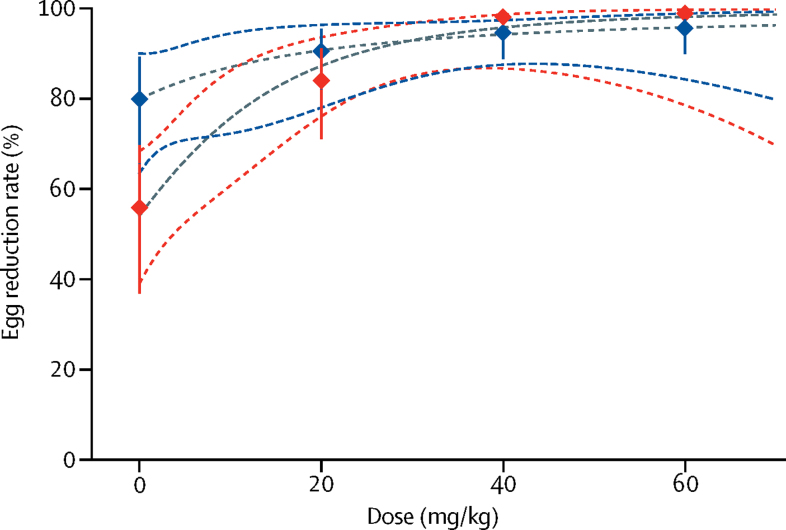
Egg reduction rates in PSAC (blue lines) and SAC (red lines) Diamonds show observed cure rates with 95% CIs (vertical lines). Dashed lines represent the estimated dose response curve and corresponding 95% confidence bands predicted by the E_max_ model. PSAC=preschool-aged children. SAC=school-aged children.

In PSAC, based on the Kato-Katz technique, both cure rates and ERRs increased only incrementally starting at 20 mg/kg. In more detail, cure rate and ERR (geometric mean) for the 20 mg/kg group were 62·1% (95% CI 44·8–77·5) and 90·7% (82·0–95·7), respectively. In the 40 mg/kg and 60 mg/kg treatment groups, similar cure rates (72·2% [95% CI 54·8–85·8] and 71·4% [53·7–85·4], respectively) were observed (table 2; figure 2). The corresponding ERRs (geometric mean) were 94·8% (95% CI 89·1–98·0) and 95·8% (90·2–98·5), respectively. PSAC in the placebo group, *S mansoni* eggs were not detected in a proportion of 37·1% (95% CI 21·5–55·1) of children with a corresponding ERR of 80·1% (66·3–88·9). The E_max_ model predicted that in PSAC, an ERR of 99% is out of the observed range, but an ERR of 95% is estimated at 50 mg/kg (figure 3). ERRs based on arithmetic means are shown in table 2.

The observed cure rates in SAC increased with escalating dosages (table 2). The 20 mg/kg dose resulted in cure in 14 (30·4%; 95% CI 17·7–45·8) children, the 40 mg/kg resulted in cure of 31 (68·9%; 53·4–81·8) children, and the 60 mg/kg dose resulted in cure of 34 (82·9%; 67·9–92·8) children based on the Kato-Katz method. Egg reduction rates (geometric mean) were moderate for the 20 mg/kg dose (84·2% [95% CI 70·9–91·5]) and high for the 40 mg/kg and 60 mg/kg treatment groups (98·3% [96·7–99·3]) and 99·1% [97·9–99·8], respectively. In the SAC in the placebo group, we observed a cure rate of 11·9% (95% CI 4·8–25·6) and an ERR of 56·0% (36·9–69·7). ERRs based on arithmetic means are shown in table 2. The E_max_ model predicted an ERR of 99·0% at 65 mg/kg in SAC (figure 3).

Cure rates in PSAC were lower based on the POC-CCA cassette test compared with the Kato-Katz technique. Considering “traces” as a positive result revealed a slightly higher cure rate at 40 mg/kg and 60 mg/kg (32·0% [95% CI 14·9–53·5]) and (33·3% [15·6–55·3]) compared with 20 mg/kg (15·4% [4·4–34·9]) and placebo (21·1% [8·3–40·9]). Considering “traces” as a negative result yielded cure rates from 21·7% (95% CI 7·5–43·7; 20 mg/kg) to 52·2% (30·6–73·2; 60 mg/kg). The POC-CCA confirmed the dose–response in SAC (table 2). Considering “traces” as a positive result revealed cure rates ranging from 19·0–55·0% for 20–60 mg/kg praziquantel and a cure rate of 10·8 in placebo treated SAC. Considering “traces” as a negative result yielded cure rates from 12·1% (placebo) to 28·9% (20 mg/kg) to 65·7% (60 mg/kg).

Imputation of missing data with treatment failure or success in the intention-to-treat analysis did not change the observed outcomes (appendix p 3). Although our study was not powered to detect differences in sex, our study did not show an effect of sex on the efficacy of praziquantel (data not shown).

Three children in the 60 mg/kg treatment group were wrongly dosed: one received a dose of 16·8 mg/kg (PSAC), one a dose of 74·5 mg/kg (PSAC), and one a dose of 36·1 mg/kg (SAC). Two PSAC in the 40 mg/kg treatment group were treated with 60·8 mg/kg and 26·3 mg/kg, respectively (figure 1). Actual doses given in PSAC were 15·2–37·5 mg/kg (20 mg/kg), 24·4–60·8 (40 mg/kg), and 36·1–72·0 mg/kg (60 mg/kg). SAC doses ranged from 13·5–28·7 mg/kg (20 mg/kg dose), 33·1–49·5 mg/kg (40 mg/kg), and 51·4–74·5 (60 mg/kg). The E_max_ model based on actual doses is shown in the appendix (p 4). The E_max_ model based on actual doses also showed a flat dose response for PSAC, whereas the response for SAC showed increasing efficacy at escalating dosages.

In an assessment of the cure rates for each *S mansoni* infection intensity category, we found that cure rates decreased as the infection intensity increased (table 2) in both age groups (PSAC or SAC) and at all praziquantel doses (appendix p 5).

Table 3 shows the number of treated children with adverse events and their dynamics over time. Adverse events data were available for 124 PSAC and 177 SAC. In the PSAC group, 30 children were absent (placebo [n=9], 20 mg/kg [n=5], 40 mg/kg [n=9], and 60 mg/kg [n=7]) following treatment and were not assessed for adverse events. Before treatment, overall 78 (63%) of 124 PSAC and 104 (59%) of 177 SAC reported mild symptoms. The recorded number of complaints was similar among treatment groups, with stomach ache and coughing most frequently reported. At 3 h post treatment, PSAC reported fewer symptoms compared with pre-treatment. In SAC there were slightly more reported episodes compared with pre-treatment at this timepoint. Overall, 29 (23%) of 124 PSAC and 124 (70%) of 177 SAC reported adverse events 3 h post treatment. Most adverse events were mild (29 [91%] of 32 episodes in PSAC and 124 [98%] of 126 episodes in SAC). No serious adverse events were reported. For both age groups, the number of adverse events was similar among the three praziquantel treatment groups, with fewer adverse events observed in the placebo groups. The most common adverse events in PSAC 3 h post treatment were diarrhoea (11 [9%] of 124) and stomach ache (ten [8%] of 124). Adverse events commonly observed in SAC were stomach ache (66 [37%] of 177), diarrhoea (50 [28%] of 177), and headache (27 [15%] of 177; table 3).

**Table 3:** Main type of clinical symptoms before treatment and adverse events 3 h and 24 h after praziquantel administration in SAC (n=177) and PSAC (n=124)

	**Preschool-aged children (PSAC)**				**School-aged children (SAC)**					
	Placebo (n=27*)	Praziquantel 20 mg/kg (n=35*)	Praziquantel 40 mg/kg (n=29*)	Praziquantel 60 mg/kg (n=33*)	Overall (n=124)	Placebo (n=42)	Praziquantel 20 mg/kg (n=47)	Praziquantel 40 mg/kg (n=46)	Praziquantel 60 mg/kg (n=42)	Overall (n=177)
**Before treatment**										
Moderate	0	0	0	0	0	0	0	0	0	0
Mild	19 (70%)	21 (60%)	17 (59%)	21 (64%)	78 (63%)	22 (52%)	29 (62%)	28 (61%)	25 (60%)	104 (59%)
None	8 (30%)	14 (40%)	12 (41%)	12 (36%)	46 (37%)	20 (48%)	18 (38%)	18 (39%)	17 (40%)	73 (41%)
Stomach ache	3 (11%)	3 (9%)	8 (28%)	4 (12%)	18 (15%)	12 (29%)	6 (13%)	10 (22%)	8 (19%)	36 (20%)
Cough	9 (33%)	11 (31%)	10 (35%)	6 (18%)	36 (2%9)	10 (24%)	22 (47%)	20 (43%)	14 (33%)	66 (37%)
Diarrhoea	6 (22%)	6 (17%)	4 (14%)	5 (15%)	21 (17%)	4 (10%)	2 (4%)	7 (15%)	0	13 (7%)
Headache	0	2 (6%)	0	1 (3%)	3 (2%)	5 (12%)	5 (11%)	1 (2%)	6 (14%)	17 (10%)
Vomiting	2 (7%)	0	3 (10%)	1 (3%)	6 (5%)	2 (5%)	2 (4%)	1 (2%)	2 (5%)	7 (4%)
Itching	2 (7%)	2 (6%)	2 (7%)	4 (12%)	10 (8%)	1 (2%)	3 (6%)	2 (4%)	1 (2%)	7 (4%)
Fever	3 (11%)	7 (20%)	7 (24%)	11 (33%)	28 (23%)	0	0	1 (2%)	2 (5%)	3 (2%)
**3 h post treatment**										
Moderate	0	1 (3%)	2 (7%)	0	3 (2%)	1 (2%)	0	0	1 (2%)	2 (1%)
Mild	3 (11%)	7 (20%)	8 (28%)	11 (33%)	29 (23%)	20 (48%)	36 (77%)	36 (78%)	32 (76%)	124 (70%)
None	24 (89%)	27 (77%)	19 (66%)	22 (67%)	92 (74%)	21 (50%)	11 (23%)	10 (22%)	9 (21%)	51 (29%)
Stomach ache	0	1 (3%)	5 (17%)	4 (12%)	10 (8%)	11 (26%)	22 (47%)	17 (37%)	16 (38%)	66 (37%)
Cough	0	2 (6%)	2 (7%)	2 (6%)	6 (5%)	3 (7%)	8 (17%)	4 (9%)	9 (21%)	24 (14%)
Diarrhoea	1 (4%)	5 (14%)	2 (7%)	3 (9%)	11 (9%)	5 (12%)	12 (26%)	17 (37%)	16 (38%)	50 (28%)
Headache	1 (4%)	1 (3%)	1 (3%)	0	3 (2%)	4 (10%)	7 (15%)	5 (11%)	11 (26%)	27 (15%)
Vomiting	0	1 (3%)	1 (3%)	2 (6%)	4 (3%)	2 (5%)	7 (15%)	9 (20%)	8 (19%)	26 (15%)
Itching	0	0	0	0	0	1 (2%)	3 (6%)	2 (4%)	1 (2%)	7 (4%)
Fever	1 (4%)	1 (3%)	0	0	2 (2%)	2 (5%)	2 (4%)	0	1 (2%)	5 (3%)
**24 h post treatment**										
Moderate	0	0	0	0	0	0	0	0	0	0
Mild	4 (15%)	5 (14%)	1 (3%)	6 (18%)	16 (13%)	16 (38%)	17 (36%)	18 (39%)	16 (38%)	67 (38%)
None	23 (85%)	30 (86%)	28 (97%)	27 (82%)	108 (87%)	26 (62%)	30 (64%)	28 (61%)	26 (62%)	110 (62%)
Stomach ache	0	0	0	0	0	7 (17%)	9 (19%)	4 (9%)	8 (19%)	28 (16%)
Cough	0	0	0	2 (6%)	0	5 (12%)	5 (11%)	5 (11%)	5 (12%)	20 (11%)
Diarrhoea	0	5 (14%)	0	1 (3%)	6 (5%)	5 (12%)	4 (9%)	3 (7%)	6 (14%)	18 (10%)
Headache	1 (4%)	0	1 (3%)	1 (3%)	3 (2%)	2 (5%)	2 (4%)	3 (7%)	4 (10%)	11 (6%)
Vomiting	2 (7%)	1 (3%)	0	1 (3%)	4 (3%)	2 (5%)	2 (4%)	3 (7%)	4 (10%)	11 (6%)
Itching	0	0	0	1 (3%)	0	4 (10%)	3 (6%)	3 (7%)	3 (7%)	13 (7%)
Fever	1 (4%)	0	0	1 (3%)	2 (2%)	1 (2%)	0	0	0	1 (1%)

Data are n (%).

* 30 kids were absent (placebo [n=9], 20 mg/kg [n=5], 40 mg/kg [n=9], and 60 mg/kg [n=7]) following treatment and were not assessed for adverse events.

24 h post-treatment, 16 episodes (13%) were noted by PSAC and 67 (38%) episodes by SAC. All reported adverse events were mild. No difference in the number of adverse events was noted among the different treatment groups and placebo group. Diarrhoea (six [5%] of 124) and vomiting (four [3%] of 124) were the two most common symptoms in PSAC 24 h post-treatment. Stomach ache (28 [16%] of 177), cough (20 [11%] of 177), and diarrhoea (18 [10%] of 177) were most frequently observed in SAC. Adverse events observed 48 h and 72 h post treatment are presented in the appendix p (6).

## Discussion

In the absence of a paediatric formulation of praziquantel, the accurate management of young children with schistosomiasis at point-of-care and at community levels may be challenging. Yet, by focusing treatment in preventive chemotherapy programmes on the school-aged population, children of preschool age are neglected, thus preventing them from benefiting from treatment given to their older peers, and hence creating a potential health inequity. If children as young as age 2 years can be infected (as shown in ours and previous studies),^12^, ^22^ but must wait until school age to be treated, they are probably already facing consequences of the chronic infection at a young age, which might carry on into older age. At the moment, praziquantel is widely used off-label at a standard dose of 40 mg/kg to treat PSAC, because this is the dose used for SAC and adults.^25^, ^28^, ^29^, ^30^ However, the effective dose for children younger than 4 years is unknown. Although the efficacy and safety of praziquantel has been intensively assessed in SAC,^28^, ^29^, ^30^ and more recently in PSAC,^31^, ^32^, ^33^, ^34^ surprisingly, well designed dose–response relation studies are lacking in both age groups.

Our randomised controlled trial, assessing the efficacy and safety of 20 mg/kg, 40 mg/kg, and 60 mg/kg praziquantel in PSAC and SAC to uncover the nature of the dose-related effect, aimed to fill this gap. We used baseline and follow-up surveys at a reasonably sensitive diagnostic approach aiming for four Kato-Katz thick smears for the diagnosis of *S mansoni* in all study participants. Additionally, a urine POC-CCA cassette test was used for diagnosis of *S mansoni*.

In SAC, an increasing efficacy was noted with escalating doses of praziquantel. The highest cure rate and ERR (geometric mean) were observed with 60 mg/kg praziquantel. The E_max_ model predicted an ERR of 99·0% at 65 mg/kg in SAC (figure 3). 60 mg/kg showed a slightly better performance than did 40 mg/kg praziquantel in terms of cure rate and ERR; however, the overlapping confidence intervals suggest that both doses perform similarly. Our findings are in line with a recent meta-analysis, which reported a cure rate of 74·6% (95% CI 68·3–80·6) using 40 mg/kg praziquantel to treat *S mansoni* infections in SAC, and a significant relation between the cure rates and dose in the treatment of *S mansoni.*^27^ In addition, a multicentre study comparing the efficacy of 40 mg/kg versus 60 mg/kg praziquantel concluded that the higher dose offers no significant efficacy advantage over the standard dose.^28^

We did not identify a no-effect dose range in PSAC. Moderate cure rate and ERR were observed with 20 mg/kg (cure rate of 62·1%; ERR of 90·7%), which slightly increased when 40 mg/kg (cure rate of 72·2%; ERR of 94·8%) or 60 mg/kg (cure rate of 71·4%; ERR of 95·8%) were given. The E_max_ model predicted that in PSAC an ERR of 99% is out of the observed range. If we calculate arithmetic means, then ERRs in PSAC ranged from 80·9–84·1%. Hence, cure rates and ERRs were lower at 40 mg/kg and 60 mg/kg praziquantel in PSAC compared with in SAC (regardless of diagnostic tool). Notably, recent WHO Standard Operating Procedures suggested a reference drug efficacy value of 90% or higher based on arithmetic means for treating *S mansoni* infections with praziquantel.^35^ All doses investigated in PSAC—namely, 20 mg/kg, 40 mg/kg, and 60 mg/kg—would therefore not fulfil the criteria of clinical efficacy. Enzymatic processes and the immune system have been well documented to be age-dependent and might affect the absorption, distribution, metabolism, and elimination of drugs in young children.^18^, ^20^, ^21^, ^36^ Because SAC were treated side-by-side with the same praziquantel dosages, in the same ecological setting (villages were all within a radius of 5 km), using the same diagnostic tests, and administering the same food item, confounding factors can be ruled out. However, crushing the tablets might have altered the bioavailability and pharmacokinetic properties of praziquantel and might therefore play a part in the difference in efficacy noted in PSAC.

The efficacy observed for 40 mg/kg in PSAC corresponds with results reported by the above-mentioned meta-analysis,^27^ which calculated a cure rate of 69% and an ERR of 85·6% for this praziquantel dose in PSAC. A recent study in a small cohort in Ugandan children showed higher cure rates and ERRs with 60 mg/kg praziquantel (ERR 91%, cure rate 82%) versus 40 mg/kg (ERR 82%, cure rate 70%),^34^ however two-thirds of the examined children were school-aged and hence no real conclusion on the efficacy of praziquantel on young children could be drawn.

One limitation of our study was that, based on the tablet formulation used in the current study (600 mg praziquantel tablets; Cesol), only half tablets increments (ie, 300 mg) could be given. Using tablets, which could have been divided in four parts (ie, Distocide) could have allowed us to dose slightly more accurately. As proposed recently by Olliaro and colleagues in 2013,^31^ we strongly recommend that when working with PSAC, a formulation that can be divided into four parts (to give 150 mg increments) be used, particularly in children weighing less than 10 kg as long as a novel paediatric formulation based on oral dispersible tablets is under development.

Another limitation of our study is a high proportion of PSAC treated with placebo showing no eggs at the follow-up using the Kato-Katz technique. The Kato-Katz method has been well documented to be less sensitive, especially in populations with low infection intensity such as PSAC or in communities having received treatment. In our study, most PSAC had a light infection intensity. 45 PSAC (about 14%) had only one single egg found in all four Kato-Katz thick smears taken together at baseline. In 2015, Siqueira and colleagues^37^ assessed parasitological and molecular techniques for the diagnosis and assessment of cure in individuals infected with *S mansoni* harbouring a light infection and concluded that an increased number of Kato-Katz slides or a test with higher sensitivity is required for participants with a very low parasite load situation, such as after therapeutic interventions. Our study therefore included the sensitive POC-CCA cassette test as an alternative diagnostic instrument. As expected, cure rates were significantly lower using the POC-CCA compared with the Kato-Katz, but across all treatment groups, due to the higher sensitivity of this device.^38^ However, this finding does not only point to treatment failures of chronic *S mansoni* infections. Indeed, praziquantel is largely refractory against young developing stages of the worms,^14^ and hence antigens might be present in the urine of young children due to acute infections contributing to the low cure rates observed with POC-CCA in this study.

The nature of the dose–response relation based on POC-CCA were similar to the one observed with Kato-Katz in SAC. Regardless of whether traces were considered as positive or negative, a dose–response relation was observed in SAC with the highest efficacy observed for SAC at 60 mg/kg (55·0 and 65·7% cure rate, respectively). In PSAC, similar cure rates were observed at 40 mg/kg and 60 mg/kg considering traces as positive (32·0% and 33·3%), which were higher than the ones recorded for 20 mg/kg (cure rate of 15·4%). However, when considering traces as negative, increasing cure rates were observed with increasing dosages in this group (21·7–52·2%). Of note, debate is still ongoing about the correct interpretation to give to POC-CCA “trace” scores in the diagnosis of *S mansoni*,^39^ hence the Kato-Katz diagnosis remains the diagnostic of choice at the moment, also for regulators.

We found that praziquantel at the investigated doses, was well tolerated by SAC and PSAC with only mild adverse events observed over time. Almost all adverse events disappeared within the 24 h following praziquantel administration. Our findings are in line with previous studies assessing safety of praziquantel in SAC^28^, ^29^, ^30^ and PSAC.^31^, ^32^, ^33^ Several issues are worth highlighting. First, fewer adverse events were noted in the placebo group compared with the praziquantel groups for both age groups. Second, no statistical difference was observed in the number of adverse events between treatment groups. Praziquantel shows good tolerability even at the highest dosage of 60 mg/kg. Third, although the frequency of adverse events peaked at 3 h post treatment in SAC; in PSAC, fewer clinical symptoms were reported 3 h post treatment compared with symptoms before treatment. However, we note that mothers were acting on behalf of their children in the statement of adverse events which could strongly influence this result.

In conclusion, praziquantel shows lower efficacies in PSAC compared with in SAC with none of the doses achieving a satisfactory ERR based on WHO guidelines^34^ and moderate cure rates observed at all doses. However, in the absence of alternative treatments, a single dose of praziquantel (40 mg/kg), as recommended by WHO can be recommended for both *S mansoni* infections in PSAC and SAC in preventive chemotherapy programmes.
