# Supplementary material for: Efficacy and safety of praziquantel in preschool-aged and school-aged children infected with Schistosoma mansoni: a randomised controlled, parallel-group, dose-ranging, phase 2 trial
Source: Lancet Glob Health. 2017 Jun 12;5(7):e688–98. doi: 10.1016/S2214-109X(17)30187-0 (PMC5471607; doi:10.1016/S2214-109X(17)30187-0)
Supplement: Supplementary appendix [file mmc1.pdf]

THE LANCET
Global Health

Supplementary appendix

This appendix formed part of the original submission and has been peer reviewed. We post it as supplied by the authors.

Supplement to: Coulibaly JT, Panic G, Silué KD, Kovač J, Hattendorf J, Keiser J.
Efficacy and safety of praziquantel in preschool-aged and school-aged children infected with *Schistosoma mansoni*: a randomised controlled, parallel, dose-finding trial.
Lancet Glob Health 2017; **5:** e688–98.

Efficacy and safety of praziquantel in school-aged and preschool-aged children infected with *Schistosoma mansoni:* a randomized controlled dose-finding trial

Jean T. Coulibaly, Gordana Panic, Dieudonné K. Silué, Jana Kovač, Jan Hattendorf, Jennifer Keiser

Department of Medical Parasitology and Infection Biology, Swiss Tropical and Public Health Institute, University of Basel, Basel, Switzerland (J T. Coulibaly PhD, G Panic MSc, J Kovac MSc, Prof J Keiser PhD); **Unité de Formation et de Recherche Biosciences, Université Félix Houphouët-Boigny, Abidjan, Côte d'Ivoire** (D K. Silué PhD); **Department of Epidemiology and Public Health, Swiss Tropical and Public Health Institute, Basel, Switzerland** (J Hattendorf PhD)

Table of contents

Table S1: Intention-to-treat analysis Page 3

Figure S1 Emax model predicting cure rates Page 4

Figure S2 Predicted probability of being cured by baseline infection intensity Page 5

Table S2: Percentage and main type of adverse events 48 and 72 hours after praziquantel administration Page 6

Table S1: Intention-to-treat analysis of dropout individuals in 20, 40 and 60 mg/kg praziquantel and placebo treatment arms among intestinal schistosomiasis infected preschool-aged children (PSAC) and school-aged children (SAC) based on Kato-Katz

	**Preschool-aged children (PSAC)**				**School-aged children (SAC)**			
	Placebo	20 mg/kg	40 mg/kg	60 mg/kg	Placebo	20 mg/kg	40 mg/kg	60 mg/kg
All dropouts cured	40.5	65.0	73.7	74.4	NA	31.9	69.6	83.3
None of the dropouts cured	35.1	57.5	68.4	64.1	NA	29.8	67.4	81.0

NA not available since no dropouts

Figure S1 Emax model predicting cure rates (CRs) in relationship to drug per kg body weight in preschool-aged children (blue symbols) and school-aged children (red symbols). Gray scale and numbers represent geometric mean infection intensity at baseline. The diameter of the symbols indicates the number of participants in each category.

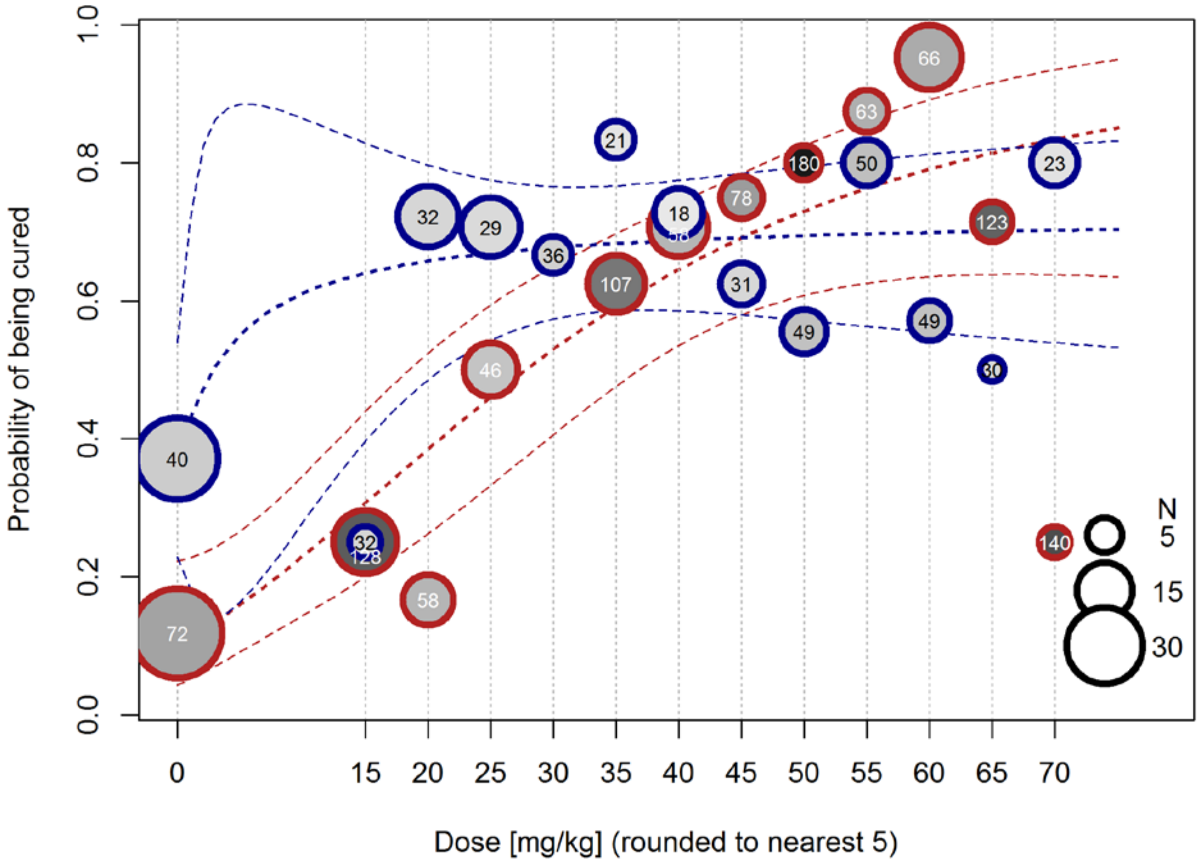

Figure S2 Predicted probability of being cured by baseline infection intensity in pre-school children (blue symbols) and school age children (red symbols). Lines represent the predicted probabilities for the 8 treatment arms estimated by logistic regression. Circles represent predicted values of participants cured after treatment, stars indicate participants remained infected after treatment. Darker symbols indicate higher infection intensity at follow up. Small random noise has been added to avoid overplotting.

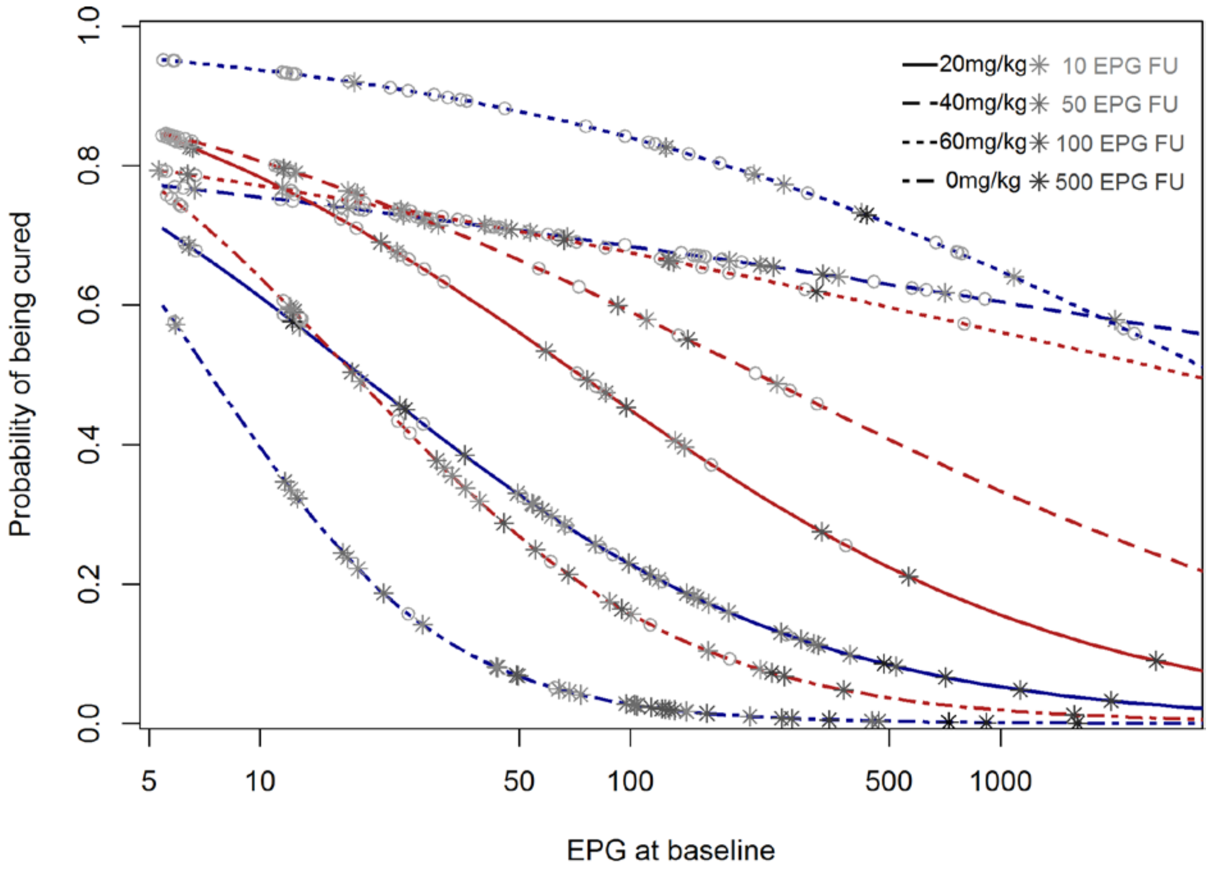

Table S3: Percentage and main type of adverse events 48 and 72 hours after praziquantel administration in school-aged children (***n* = 177**) and preschool-aged children (***n* = 124**)

	**Preschool-aged children (PSAC)**					**School-aged children (SAC)**				
**Symptoms**	Placebo **(n = 27*)**	20 mg/kg **(n = 35*)**	40 mg/kg **(n = 29*)**	60 mg/kg **(n = 33*)**	Overall **(n=124)**	Placebo **(n= 42)**	20 mg/kg **(n = 47)**	40 mg/kg **(n = 46)**	60 mg/kg **(n = 42)**	Overall **(n=177)**
**48 h posttreatment**										
Moderate	0 (0)	0 (0)	0 (0)	0 (0)	0 (0)	0 (0)	0 (0)	0 (0)	0 (0)	0 (0)
Mild	2 (7)	4 (11)	5 (17)	4 (12)	15 (12)	13 (31)	13 (28)	12 (26)	12 (29)	50 (28)
None	25 (93)	31 (86)	24 (83)	29 (88)	109 (88)	29 (69)	34 (72)	34 (74)	30 (71)	127 (72)
Stomach ache	0 (0)	0 (0)	0 (0)	1 (3)	1 (1)	5 (12)	6 (13)	6 (13)	4 (10)	21 (12)
Cough	1 (4)	0 (0)	3 (10)	1 (3)	5 (4)	4 (10)	4 (9)	6 (13)	6 (14)	20 (11)
Diarrhoea	1 (4)	3 (9)	0 (0)	1 (3)	6 (5)	2 (5)	1 (2)	1 (2)	3 (7)	7 (4)
Headache	0 (0)	0 (0)	0 (0)	0 (0)	0 (0)	2 (5)	2 (4)	2 (5)	3 (7)	6 (3)
Vomiting	0 (0)	0 (0)	0 (0)	0 (0)	0 (0)	0 (0)	0 (0)	1 (2)	2 (5)	3 (2)
Itching	0 (0)	1 (3)	0 (0)	1 (3)	2 (2)	4 (10)	3 (6)	2 (5)	1 (2)	10 (6)
Fever	2 (7)	0 (0)	2 (7)	0 (0)	4 (3)	1 (2)	0 (0)	0 (0)	0 (0)	1 (1)
**72 h posttreatment**										
Moderate	0 (0)	0 (0)	0 (0)	0 (0)	0 (0)	0 (0)	0 (0)	0 (0)	0 (0)	0 (0)
Mild	1 (4)	0 (0)	0 (0)	1 (3)	2 (2)	9 (21)	13 (29)	13 (28)	13 (30)	48 (27)
None	26 (96)	35 (100)	29 (100)	32 (97)	122 (98)	33 (79)	32 (71)	34 (72)	30 (70)	129 (73)
Stomach ache	0 (0)	0 (0)	0 (0)	0 (0)	0 (0)	4 (10)	6 (13)	4 (9)	4 (9)	18 (10)
Cough	0 (0)	0 (0)	0 (0)	0 (0)	0 (0)	4 (10)	3 (6)	8 (17)	8 (19)	23 (13)
Diarrhoea	1 (4)	0 (0)	0 (0)	0 (0)	1 (1)	0 (0)	1 (2)	1 (2)	3 (7)	5 (3)
Headache	0 (0)	0 (0)	0 (0)	0 (0)	0 (0)	2 (5))	3 (6)	2 (4)	4 (10)	11 (6)
Vomiting	0 (0)	0 (0)	0 (0)	0 (0)	0 (0)	0 (0)	0 (0)	1 (2)	0 (0)	1 (1)
Itching	0 (0)	0 (0)	0 (0)	0 (0)	0 (0)	3 (7)	2 (4)	1 (2)	2 (5)	8 (5)
Fever	0 (0)	0 (0)	0 (0)	1 (3)	1 (1)	1 (2)	0 (0)	1 (2)	0 (0)	2 (1)

*30 kids were

absent [placebo (n=9), 20 mg/kg (n=5), 40 mg/kg (n=9) and 60 mg/kg (n=7)] following treatment and were not assessed for adverse events
